# The invariant arginine within the chromatin-binding motif regulates both nucleolar localization and chromatin binding of Foamy virus Gag

**DOI:** 10.1186/s12977-018-0428-z

**Published:** 2018-07-11

**Authors:** Joris Paris, Joëlle Tobaly-Tapiero, Marie-Lou Giron, Julien Burlaud-Gaillard, Florence Buseyne, Philippe Roingeard, Pascale Lesage, Alessia Zamborlini, Ali Saïb

**Affiliations:** 1 CNRS UMR7212, Hôpital St Louis, Inserm U944, Institut Universitaire d’Hématologie, Université Paris Diderot, Sorbonne Paris Cité, Paris, France; 2 Plateforme IBiSA de Microscopie Electronique, Université François Rabelais and CHRU de Tours, Tours, France; 3 INSERM U1259, Université François Rabelais and CHRU de Tours, Tours, France; 4 Institut Pasteur, Unité d’Epidémiologie et Physiopathologie des Virus Oncogènes, Paris, France; 5 CNRS UMR3569, Insitut Pasteur, Paris, France; 6 CNRS UMR7212, Hôpital St Louis, Inserm U944, Institut Universitaire d’Hématologie, Université Paris Diderot, Sorbonne Paris Cité, Laboratoire PVM, Conservatoire National des Arts et Métiers (Cnam), Paris, France

**Keywords:** Foamy virus, Gag, Nuclear trafficking, Nucleolus, Chromatin-binding, Post-translational modification, Methylation, PRMT

## Abstract

**Background:**

Nuclear localization of Gag is a property shared by many retroviruses and retrotransposons. The importance of this stage for retroviral replication is still unknown, but studies on the Rous Sarcoma virus indicate that Gag might select the viral RNA genome for packaging in the nucleus. In the case of Foamy viruses, genome encapsidation is mediated by Gag C-terminal domain (CTD), which harbors three clusters of glycine and arginine residues named GR boxes (GRI-III). In this study we investigated how PFV Gag subnuclear distribution might be regulated.

**Results:**

We show that the isolated GRI and GRIII boxes act as nucleolar localization signals. In contrast, both the entire Gag CTD and the isolated GRII box, which contains the chromatin-binding motif, target the nucleolus exclusively upon mutation of the evolutionary conserved arginine residue at position 540 (R540), which is a key determinant of FV Gag chromatin tethering. We also provide evidence that Gag localizes in the nucleolus during FV replication and uncovered that the viral protein interacts with and is methylated by Protein Arginine Methyltransferase 1 (PRMT1) in a manner that depends on the R540 residue. Finally, we show that PRMT1 depletion by RNA interference induces the concentration of Gag C-terminus in nucleoli.

**Conclusion:**

Altogether, our findings suggest that methylation by PRMT1 might finely tune the subnuclear distribution of Gag depending on the stage of the FV replication cycle. The role of this step for viral replication remains an open question.

**Electronic supplementary material:**

The online version of this article (10.1186/s12977-018-0428-z) contains supplementary material, which is available to authorized users.

## Background

Foamy viruses (FVs), also known as spumaviruses, are complex retroviruses that belong to the *Spumaretrovirinae* subfamily of the Retroviridae. They are endemic among many animal species, particularly non-human primates (NHPs) (for a review [1]). The Prototype FV (PFV) was isolated from human-derived cell culture and later found to be a chimpanzee FV [2]. It is currently well established that humans are not natural hosts but acquire infection as a consequence of zoonotic transmission of simian FVs (SFVs) through bites of captive or wild NHPs [3, 4]. FV infection is persistent and apparently benign [1] and human-to-human transmission has never been reported. Like all retroviruses, FVs reverse transcribe their RNA genome (gRNA), which encodes the typical *gag*, *pol* and *env* genes, and integrate the resulting cDNA into the host cell chromosomes. However, specificities in the replication strategy of FVs set them apart from orthoretroviruses. These include the fact that reverse transcription occurs during viral particle production [5, 6], and that Pol is expressed independently of Gag from a specific spliced transcript [7, 8]. The structural organization and maturation profile of FV Gag are also peculiar. FV Gag lacks the major homology region (MHR) and the Cys-His zinc-finger motifs that are hallmarks of orthoretroviral Gag proteins. Moreover, FV Gag is not processed into the matrix (MA), capsid (CA) and nucleocapsid (NC) mature products like its orthoretroviral counterparts, but rather undergoes a single cleavage event that removes a 4 kDa C-terminal peptide ([9], reviewed in [10]). This feature is shared by the Gag proteins of the *Drosophila* retrovirus Gypsy [11] and the Ty1 retrotransposon of *S. cerevisiae* [12]. Recent studies showed that PFV Gag N-terminal domain (NTD, amino acids (aa) 1–180) is entirely unrelated to its orthoretroviral counterpart [13]. They also confirmed that the NTD, which harbors the cytoplasmic targeting and retention signal (CTRS) and the self-dimerization domain, plays a role similar to orthoretroviral CA in viral capsid assembly [13]. In contrast the central conserved region of PFV Gag (aa 300–477), which is involved in the formation of higher-order multimers, shares a conformation analogous to that of orthoretroviral CA, suggesting evolution from a common ancestral protein [14]. In the absence of structural data, functional studies indicate that the C-terminal domain (CTD, aa 400–648) of FV Gag plays a role related to that of orthoretroviral NC in genome packaging [10, 15]. This domain is enriched in glycine and arginine residues that in primate FV Gag proteins are clustered in three regions named GR boxes (GRI-III) [15]. GRI binds nucleic acids in vitro and was proposed to be responsible of the incorporation of both the gRNA and Pol into virions [16–18]. The GRII box shows the highest conservation throughout evolution and is involved in the accumulation of PFV Gag in the nucleus [15]. The determinant for nuclear localization within GRII maps to a 13-aa chromatin binding sequence (CBS, aa 534–546) that recognizes the H2A/H2B core histones. This interaction tethers the pre-integration complex (PIC) to host cell chromatin prior to viral integration [19–21]. The role of GRIII in FV replication is enigmatic but likely related to that of GRI, since the two motifs can functionally complement each other [22]. Although the GR boxes were initially viewed as independent entities playing both specific and redundant functions, a recent study rather indicates that the positively charged residues within the CTD, not the GR boxes individually, mediate gRNA packaging and Pol encapsidation [23].

During FV replication Gag displays different subcellular localizations, as a result of numerous interactions with the intracellular trafficking machinery, which likely match its multiple roles throughout the viral life cycle. Our previous studies showed that, as a component of the incoming PIC, Gag drives the traffic of viral particles towards the microtubule-organizing center (MTOC) where uncoating occurs [24–26]. Upon nuclear envelope breakdown, Gag associates with host cell chromosomes and critically contributes to the selection of the integration sites [19–21, 27]. At a later stage, newly synthesized Gag molecules have been shown to oligomerize in the nucleus and, next, reach the cytoplasm by engaging the CRM1 (Chromosomal Maintenance 1, also known as Exportin 1)-dependent pathway through a nuclear export signal (NES) [28], as reported for Rous Sarcoma Virus (RSV) (see below, [29]). Currently, how newly synthesized Gag crosses the intact nuclear membrane is unknown [20].

Evidence that Gag proteins shuttle between the nucleus and the cytoplasm has been reported for other retroviruses, including RSV, feline immunodeficiency virus (FIV), Human Immunodeficiency Virus (HIV), mouse mammary tumor virus (MMTV), Mazon-Pfizer monkey virus (MPMV) and murine leukemia virus (MLV) ([30] and references therein), and the Tf1 retrotransposon [31]. Gag or the isolated NCs of MMTV, RSV, FIV, HIV and MLV have also been detected in the nucleolus ([30] and references therein), a distinct subnuclear compartment that forms around the gene clusters encoding rRNAs and represents the site of ribosomes biogenesis. Although in most instances the significance of Gag nucleocytoplasmic trafficking for virus replication is elusive, RSV Gag was shown to oligomerize in the nucleus, in an RNA- and NC-dependent manner [29]. The observation that nuclear trafficking of RSV Gag is required for efficient genome encapsidation [32, 33] and that binding to a synthetic oligonucleotide mimicking the packaging signal favours the association between Gag and the nuclear export factor CRM1 in vitro [34], led to propose a model according to which RSV Gag selects the viral gRNA for packaging in the nucleus.

To deepen our understanding of the nuclear trafficking of PFV Gag, we studied the localization of the C-terminal GR boxes and established that the isolated GRI and GRIII boxes are nucleolar localization signals (NoLSs). We also found that Gag localizes at least temporarily in the nucleolus during PFV replication. Next, we investigated the mechanisms that regulate this process, and identified the evolutionary conserved arginine residue at position 540 (R540) within the GRII box as a critical factor determining whether Gag localizes in the nucleolus or is tethered to chromatin. We also established that PFV Gag interacts with and is modified by PRMT1 (Protein Arginine Methyltransferase 1) and PRMT5. Interestingly, we found that PFV Gag harboring the R540A substitution is unable to interact with PRMT1 and lost the asymmetric dimethylarginine (ADMA) mark, while retaining binding to and modification by PRMT5. Finally, we observed that siRNA-mediated depletion of PRMT1 leads to nucleolar accumulation of the C-terminus of PFV Gag fused to GFP. On the basis of these results, we hypothesize that PRMT1-mediated methylation, which requires the invariant R540 residue, could regulate the subnuclear distribution of PFV Gag antagonizing nucleolar accumulation in favor of chromosome binding.

## Results

### The GRI and GRIII boxes of PFV Gag are nucleolar localization signals

**Fig. 1:**
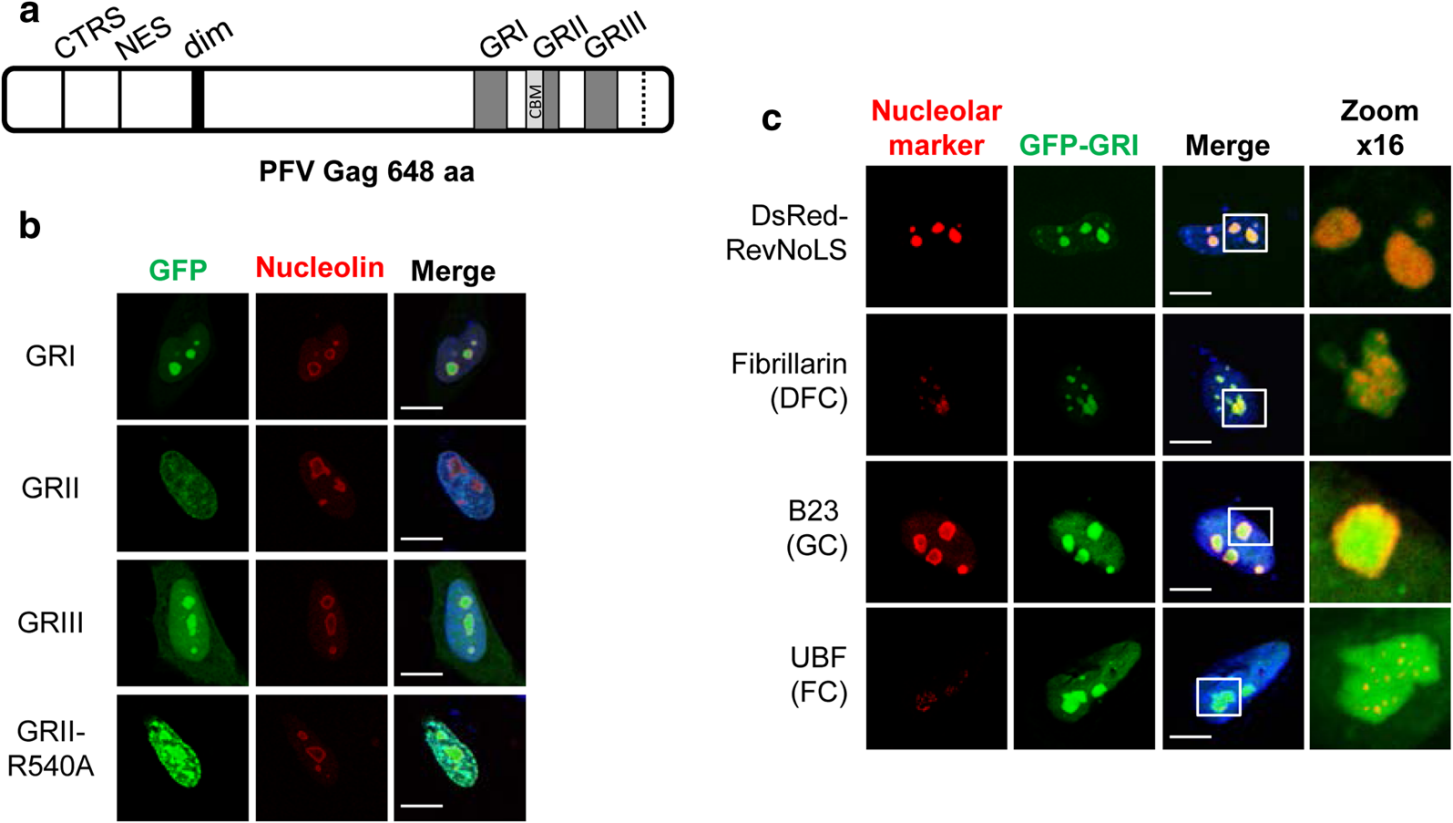
GRI and GRIII boxes of PFV Gag are Nucleolar Localization Signals. **a** Scheme of PFV Gag protein where the primary protease-cleavage site at residue 621 is indicated by a dotted line. Some characterized motifs are shown. CTRS: cytoplasmic targeting and retention signal (aa 43–60); NES: nuclear export signal (aa 95–112); dim: dimerization domain (aa 130–160); GRI, GRII and GRIII: glycine-arginine rich box I (aa 485–511), II (aa 534–557) and III (aa 586–618); CBM, chromatin-binding motif (aa 536–544). **b** The subcellular localization of GRI, GRII, GRII_R540A_ or GRIII expressed as GFP-fusion proteins in fixed HeLa cells was analyzed 24 h post-transfection by immunofluorescence and confocal microscopy. Nucleoli were immune-stained with an anti-nucleolin antibody (ab 22758, Abcam, 1:800) and nuclei were stained with DAPI (blue). **c** The localization of GFP-GRI expressed in HeLa cells relative to the NoLS of HIV-1 Rev protein (aa 35–51) in fusion with DsRed or specific markers of the nucleolar subcompartments was studied as in B. Cells were stained with antibodies against fibrillarin (c13c3, Cell signaling, 1:200), B23 (sc6013_R, Santa Cruz, 1:200) or UBF (H300, Santa Cruz, 1:200) to visualize the dense fibrillar component (DFC), the granular component (GC) and the fibrillar center (FC), respectively. The right column (zoom × 16) corresponds to the enlarged images from the boxed areas. Scale bar represents 10 µm

The GR boxes within PFV Gag CTD are short sequences enriched in arginine residues, which is a hallmark of NoLSs [35] (Fig. 1a and Additional file 1: S1A). To determine if these motifs could localize proteins to the nucleolus, we cloned each GR box in frame with the *EGFP* gene. The cellular distribution of the resulting GFP-fusion proteins was studied in HeLa cells that were stained for nucleolin, one of the most abundant proteins of the nucleolus [36]. We found that GFP-GRI was concentrated in nucleolin-positive foci, GFP-GRII displayed a diffuse nuclear staining, while GFP-GRIII was both enriched in nucleolin-positive foci and distributed throughout the nucleoplasm and the cytoplasm (Fig. 1b). GFP-GRI staining also partially overlapped with DsRed fused to the NoLS of the HIV Rev protein (DsRed-RevNoLS) (Fig. 1c), which localizes in both the Dense Fibrillar Component (DFC) and the Granular Component (GC) nucleolar compartments where the early events in rRNA transcription and processing and maturation of pre-ribosomal subunits occur, respectively [37]. In agreement with this observation, GFP-GRI co-localized with fibrillarin and nucleophosmin/B23, which specifically mark the DFC and the GC, respectively (Fig. 1c). Furthermore, an overlap between GFP-GRI and the Upstream Binding Factor (UBF) located in the Fibrillar Center (FC) was observed (Fig. 1c). Ultrastructural analysis by immunoelectron microscopy confirmed the presence of GFP-GRI and GFP-RevNoLS in the DFC and GC (Additional file 1: Fig S1B). We further observed that DsRed-GRI and GFP-GRIII co-localized when expressed in the same cell, confirming that the GRI and the GRIII box target the same subnuclear compartment (Additional file 1: Fig. S1C). A co-localization was observed between DsRed-PFV-GRI and GFP fused to either the GRI or the GRIII box of the Equine Foamy virus (EFV), the most distantly related FV (Additional file 1: Fig. S1C). Altogether these results indicate that the GRI and GRIII boxes of FV Gag proteins are NoLSs able to induce the nucleolar localization of a heterologous protein, and that this function is conserved among primate and non-primate FVs.

### FV Gag transits through the nucleolus during viral replication

**Fig. 2:**
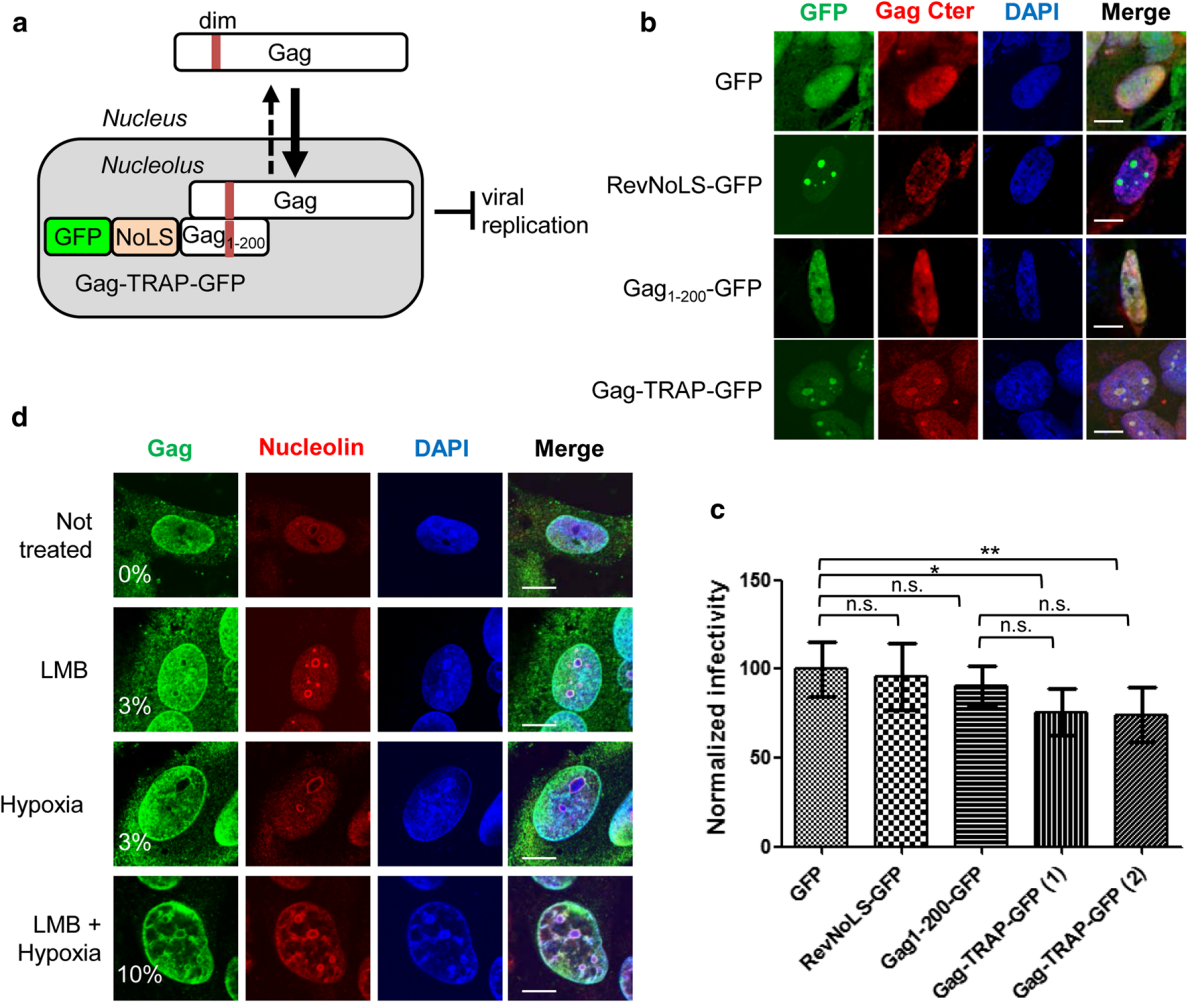
Gag transits through the nucleolus during PFV infection. **a** Schematic representation of the experimental strategy used to study PFV Gag trafficking through the nucleolus in U373MG cells stably expressing the Gag-TRAP-GFP protein (Gag_1-200-_RevNoLS-GFP). **b** U373MG cell lines stably expressing GFP, RevNoLS-GFP, Gag_1-200_-GFP or Gag-TRAP-GFP were infected with replication competent PFV. After 72 h, the localization of Gag (red staining) was analyzed in fixed cells using a rabbit polyclonal antibody specific of the C-terminal half of Gag (aa 382–648). Images were acquired as described in Fig. 1b. **c** Virions released in the supernatant 72 h after infection were titrated on FAG indicator cells and the percentage of infected (GFP-positive) cells was measured by flow cytometry. The infectivity of virions produced by GFP-expressing U373MG cells was used for normalization. Results from 4 independent experiments performed in three replicates each are expressed as the mean ± SD (standard deviation). Significance compared to GFP was calculated using a one-way ANOVA statistical test with a Bonferroni Multiple comparison post-test (*p < 0.05; **p < 0.01). **d** The subcellular localization of Gag was studied in PFV infected U373MG cells treated or not with LMB (10 nM, 6 h) and/or exposed to hypoxia (2% O_2_, 4 h). At 48 h post-infection, cells were fixed and stained with a mouse polyclonal antibody against full-length Gag (green) and rabbit polyclonal anti-nucleolin antibody (ab 22,758, Abcam, 1:800). Two hundreds cells were counted for each sample. Nuclei were stained with DAPI (blue). Images were acquired as described in Fig. 1b. Scale bar represents 10 µm

Having shown that GRI and GRIII are NoLSs, we asked whether FV Gag transits through the nucleolus during PFV life cycle. The fact that PFV Gag has never been detected in the nucleolus of infected cells suggests that either this process is highly dynamic and/or that only a small fraction of the protein resides in the nucleolus at steady state. To address this question, we adopted a “capture” assay similar to that used to demonstrate nucleolar trafficking of HIV Rev [38]. To this end, we established U373MG cells stably expressing a chimeric protein named Gag-TRAP-GFP, which consists of the N-terminal region of Gag (aa 1–200) including the dimerization domain [13, 39], fused with the NoLS of HIV Rev and GFP (Fig. 2a). We reasoned that if FV Gag transits through the nucleolus, it would interact with Gag-TRAP-GFP and be consequently retained at this site. As expected, Gag-TRAP-GFP accumulates in the nucleolus (Fig. 2b). We also confirmed that Gag-TRAP-GFP co-precipitates with full-length PFV Gag **(**Additional file 2: Fig. S2**)**. Next, U373MG cells stably expressing Gag-TRAP-GFP, or the appropriate controls (GFP, Gag_1-200_-GFP or RevNoLS-GFP), were infected with PFV. Seventy-two hours later, Gag distribution was analyzed by immunofluorescence and confocal microscopy with an antibody directed against the C-terminal half of Gag. In control cells, Gag (red staining) localized in the cytoplasm and/or in the nucleus, but was not detected in the nucleolus (Fig. 2b), while in Gag-TRAP-GFP-expressing cells, Gag was diffused in the nucleoplasm and co-localized with the chimeric protein in the nucleolus. The infectivity of viruses released in the cell culture supernatant was quantified in parallel using FAG-indicator cells [39]. Viruses produced from cells expressing RevNoLS-GFP or Gag_1-200_-GFP were not significantly less infectious compared to those produced from GFP-expressing cells, used for normalization (96% ± 14 and 91% ± 3 compared to 100 ± 15, respectively) (Fig. 2c). Expression of Gag-TRAP-GFP resulted in a reduction of infectivity of about 25% (75% ± 13 and 73% ± 11 for independent duplicate samples) (Fig. 2c). Statistical analysis shows that such decrease in infectivity is statistically significant when compared to the GFP, but not to the Gag_1-200_-GFP, sample (Fig. 2c).

Since we could not exclude that the interaction between Gag and Gag-TRAP-GFP occurs outside the nucleolus and that the Rev NoLS within the chimeric protein subsequently targets the complex to this subcellular site, we analyzed Gag localization in PFV-infected U937MG cells treated with leptomycine B (LMB), a specific inhibitor of CRM1-mediated nuclear export, and/or exposed to hypoxia, a setting that was previously shown to slow down protein trafficking [40]. Under these conditions, Gag (green staining) co-localized with nucleolin in about 3–10% of PFV-infected cells (Fig. 2d). Altogether these results indicate that Gag transits through the nucleolus during PFV replication.

### The evolutionary conserved R540 residue is critical for both nucleolar localization and chromosome tethering of PFV Gag

**Fig. 3:**
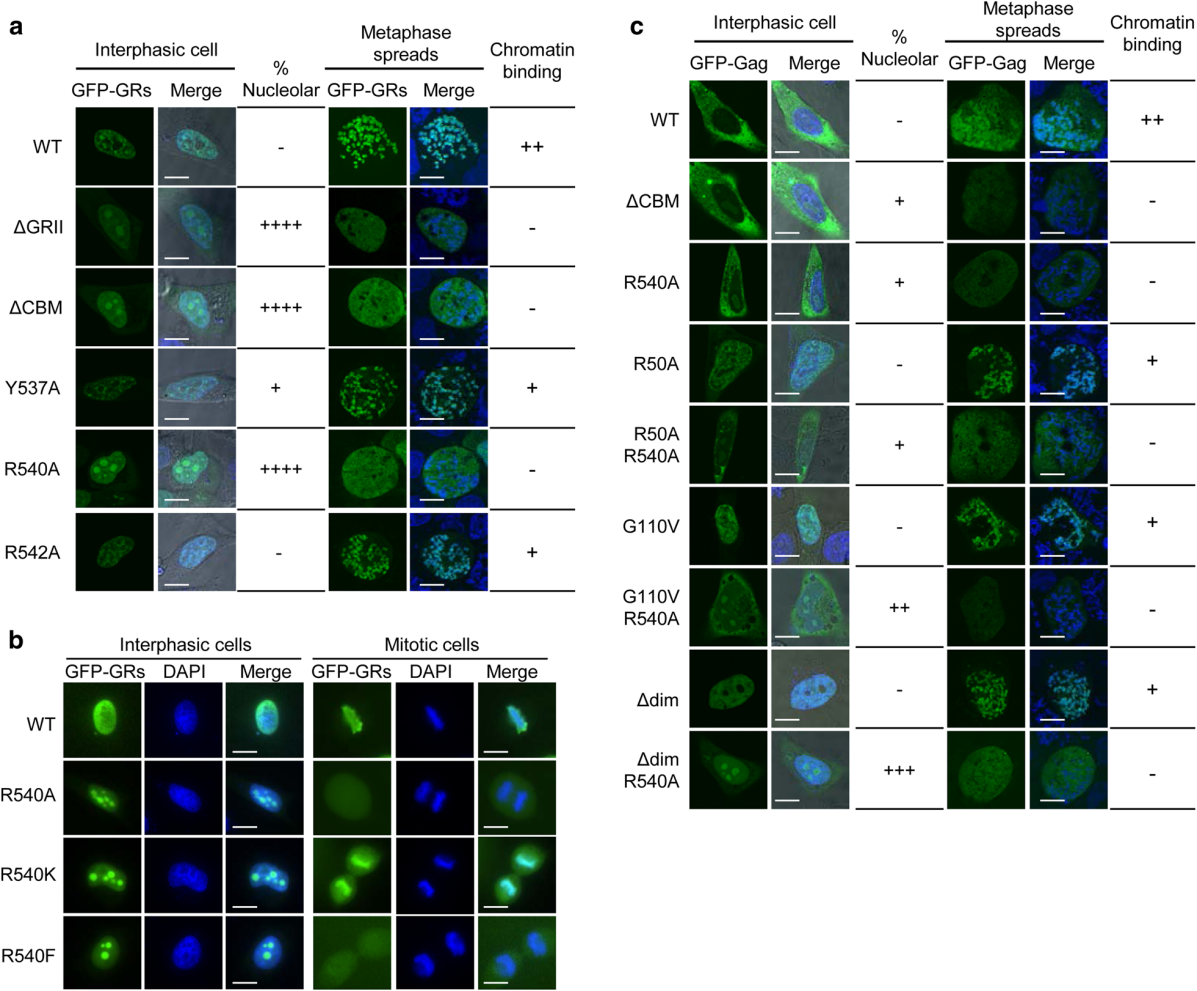
The invariant R540 residue in PFV Gag regulates nucleolar localization and binding to mitotic chromosomes. Living HeLa cells expressing the indicated GFP-GRs (**a**) or GFP-Gag (**c**) constructs and stained with Hoechst 33342 were observed on a confocal microscope 24 h after transfection (left panels). Merged images correspond to GFP, nucleic acid staining and differential interference contrast to visualize the cell shape. The “% nucleolar” column indicates the percentage of transfected cells displaying GFP staining in the nucleolus (−, < 1%; +, 1–25%; ++, 26–50%; +++, 51–75%; ++++ , 76–100%). To study the interaction of GFP-fusion proteins with chromatin (right panels), cells ectopically expressing indicated proteins were arrested in metaphase by treatment with colcemid (0.1 µg/mL, 2 h) and chromosome spreads counterstained with DAPI. Images were acquired as described in Fig. 1b. The chromatin binding column indicates whether the GFP-fusion protein was exclusively localized onto chromosomes (++), both on chromosomes and throughout the cell (+), or was distributed throughout the cell and did not associate with chromosomes (−). Representative images from two independent experiments are shown. Between 100 and 120 cells were analyzed for each condition. Scale bars represent 10 µm. **b** HeLa cells expressing the indicated GFP-GRs were stained with DAPI. Images were acquired as described in Fig. 1b. Scale bars represent 10 µm

Our observations showing that Gag localizes at least temporarily in the nucleolus during PFV infection, raise the question of how this process is regulated. Given that GRI and GRIII, but not GRII, are NoLSs (Fig. 1b), we decided first to study the subcellular distribution of GFP fused to PFV Gag CTD encompassing the three GR boxes (aa 477–625). The resulting GFP-GRs fusion protein was diffused throughout the nucleoplasm and excluded from the nucleoli (Fig. 3a, left panel), a localization pattern reminiscent of that of GFP-GRII (Fig. 1b). This observation suggested that the GRII box might antagonize the nucleolar-targeting function of GRI and/or GRIII. In support of this hypothesis, deletion of either the entire GRII box or the chromatin-binding motif (CBM, aa 536–544) in the context of GFP-GRs induced an accumulation of the corresponding mutants in the nucleolus (Fig. 3a, left panel). To map further the determinants of GRII that influence nucleolar-targeting, we aligned the sequences of the GRII box from several FV isolates and found that PFV CBM residues Y537 and R540 are strictly conserved, while R542 is only present in Gag from some primate FVs, EFV and in CoeEFV, an endogenous foamy virus-like element in the Coelacanth genome [41] (Table 1). Each of these residues was mutated within the GFP-GRs construct to address their contribution to nucleolar targeting. GFP-GRsR542A displayed a WT distribution in HeLa cells (Fig. 3a, left panel). GFP-GRsY537A localized in the nucleoplasm in most instances, but was detected also in the nucleolus in about a third of the transfected cells (Fig. 3a, left panel). In contrast, GFP-GRs where R540 is mutated to A, K or F accumulated in nucleoli in the whole population of transfected cells (Fig. 3a, left panel and Fig. 3b). These observations are consistent with the finding that GFP-GRII harboring the R540A mutation co-localizes with nucleolin (Fig. 1b). Finally, we found that GFP fused to the C-terminal region of EFV Gag (EFV GFP-GRs) was distributed in the nucleoplasm (Additional file 1: Fig. S1D). Upon alanine substitution of R472, which is equivalent of PFV Gag R540, EFV GFP-GRs accumulated in the nucleolus (Additional file 1: Fig. S1D).

**Table 1:**
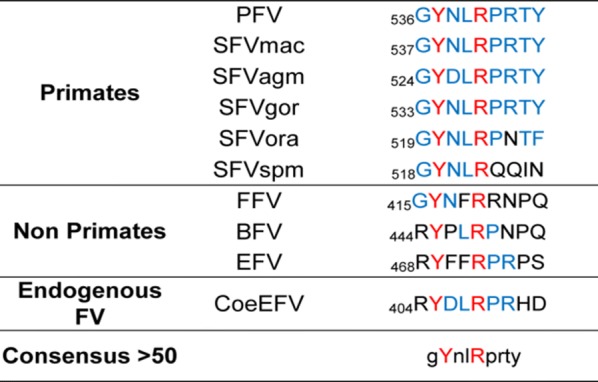
Sequence alignment of Gag CBM from different FV strains. Residues that are conserved in > 50 and 100% of the sequences are colored blue and red, respectively. The alignment was obtained using ESPript (http://espript.ibcp.fr) [58]

We next studied the influence of R540 on full-length PFV Gag localization. PFV Gag expressed as GFP-fusion protein in HeLa cells was predominantly diffused in the cytoplasm of interphasic cells, and was excluded from the nucleolus (Fig. 3c, left panel). Upon deletion of the CBM or mutation of R540 to A, GFP-Gag accumulated in the nucleolus in a fraction of transfected HeLa cells (about 7 and 10%, respectively) **(**Fig. 3c, left panel). These results contrasted with the finding that GFP-GRsR540A was detected in the nucleolus in all the transfected cells (Fig. 3a, left panel). We hypothesized that this discrepancy might result at least in part from the presence of N-terminal sequences within Gag favoring its accumulation in the cytoplasm and/or antagonizing its nuclear/nucleolar localization. To address this point, we mutated three well-characterized functional domains in GFP-Gag or GFP-GagR540A, namely the CTRS (mutation R50A) [42], the NES (mutation G110 V) [28] and the dimerization domain (Δdim, deletion of aa 130–160) [39] (Fig. 1a). In agreement with these published studies, GFP-Gag bearing the R50A or G110V or Δdim mutation accumulated in the nucleus (Fig. 3c, left panel). When any of these mutations was combined with the R540A substitution, the resulting GFP-Gag variants were distributed in the cytoplasm and the nucleoplasm and also accumulated in the nucleolus, to a variable extent (Fig. 3c, left panel).

Having previously shown that deletion of the CBM impairs FV Gag binding to mitotic chromosomes [19], we also addressed the involvement of the conserved residues within the GRII box in the interaction of FV Gag with chromatin. Gag variants where Y537 or R542 are changed to A retained the ability to interact with mitotic chromosomes (Fig. 3a, c, right panel). Notably, mutation of R540 to A was sufficient to abolish binding of either the CTD or full length Gag expressed as GFP-fusion proteins to chromatin (Fig. 3a, c, right panel). The results obtained in metaphase spreads were confirmed by the observation of fixed cells expressing GFP-GRs R540A that undergo mitosis, as judged by DNA staining (Fig. 3b). Similarly, we never observed an association between mitotic chromosomes and GFP-GRs bearing the R540F mutation, which mimics constitutive methylation (Fig. 3b). Finally, we found that GFP-GRs carrying the R540 K change painted the chromosomes of mitotic cells (Fig. 3b), indicating that a positive charge at position 540 is sufficient to ensure interaction of Gag with the H2A/H2B core histones [19, 21]. Altogether our findings indicate that the phylogenetically conserved R540 residue of PFV Gag is critical to regulate the subnuclear distribution of the protein: i.e. its nucleolar localization *versus* mitotic chromosomes binding.

### R540 is required for both PRMT1 binding and ADMA modification of PFV Gag

**Fig. 4:**
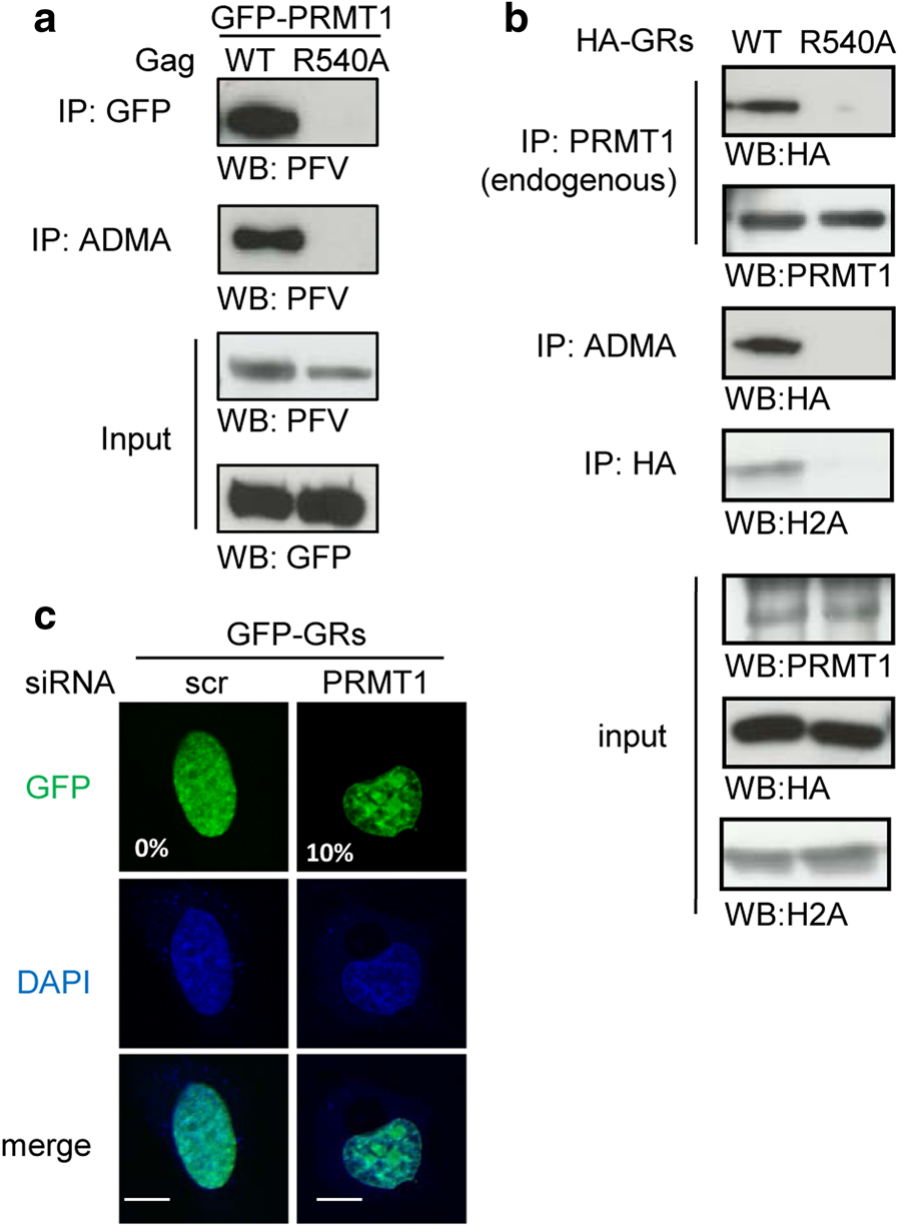
PRMT-1 binds to and methylates PFV Gag in a manner that depends on R540. **a** Following lysis, cells expressing PFV Gag WT or R450A mutant and GFP-PRMT1 were incubated with protein A beads coated with either an anti-GFP (cat.11 814 460 001, Roche, 1:100) or an anti-ADMA (ab5394 (7E6), Abcam,1:100) antibody. Input and immunoprecipitated proteins were separated by SDS-PAGE and visualized by Western blotting with anti-GFP (cat.11 814 460 001, Roche, 1:1000) or rabbit polyclonal anti-PFV antibodies. **b** Lysates from cells expressing HA-tagged GRs or GRs-R540A were immunoprecipitated with an antibody directed against PRMT1 (Cat A300-722A, Bethyl Laboratories (Euromedex), 1:100), the ADMA modification (ab5394 (7E6), Abcam, 1:100), or the HA epitope (H11, Covance, 1:100). Input and bound proteins were analyzed as in A. **c** HeLa cells were transfected with siRNA targeting PRMT1 or scrambled control (scr) and, two days later, with GFP-GRs expression plasmid. After 24 h, cells were processed as indicated in Fig. 1b. Images are representative of two independent experiments. The numbers indicate the percentage of GFP-positive cells with significant nucleolar accumulation of 100 counted cells. Scale bar represents 10 µm

To further understand the regulation of PFV Gag subnuclear distribution by R540, we asked whether this residue might be targeted by post-translational modifications, particularly methylation. To address this point, we tested whether Gag could interact with any of the nine Protein Arginine Methyltransferases identified in human cells (PRMT1 to PRMT9) [43]. We performed co-immunoprecipitation assays on 293T cells expressing WT Gag and each PRMT protein fused to GFP and observed that Gag binds to GFP-PRMT1 and GFP-PRMT5, but no other GFP-PRMTs (Additional file 3: Fig. S3A). These interactions were confirmed by performing the reciprocal experiment (Fig. 4a and Additional file 3: S3B). Interestingly, we found that the R540A Gag mutant lost the ability to interact with GFP-PRMT1 (Fig. 4a, IP GFP), but still co-precipitated with GFP-PRMT5 (Additional file 3: Fig. S3B, IP GFP). The R540A change also abolished the interaction between Gag CTD (comprising the three GR boxes) and endogenous PRMT1 (Fig. 4b, IP PRMT1). Therefore, R540 is specifically required for Gag binding to PRMT1. It is worth to mention that Gag CTD bearing the R540A substitution is unable to interact with the H2A histone (Fig. 4b, IP HA), which likely explains the impaired binding to mitotic chromosomes (Fig. 3).

PRMT1 is the primary methyltransferase that deposits the asymmetric dimethylarginine (ADMA) mark, whereas PRMT5 performs the symmetric dimethylarginine (SDMA) modification. In GFP-PRMT1-expressing cells WT Gag, but not the R540A mutant, can be precipitated with an antibody specific for the ADMA modification (Fig. 4a, IP ADMA). Similarly, WTHA-GRs, but not the R540A mutant, was enriched on beads coated with the anti-ADMA antibody (Fig. 4b). In contrast, both WT Gag and the R540A mutant co-precipitated with an anti-SDMA antibody, when expressed together with GFP-PRMT5 (Additional file 3: Fig. S3B).

Since mutation of R540 also leads to the accumulation of Gag in nucleoli, we finally asked whether PRMT1 might influence the subcellular distribution of the viral protein. To this purpose, we studied the localization of GFP-GRs in HeLa cells previously transfected with siRNA targeting PRMT1 or the appropriate scrambled control. As shown in Fig. 4c, GFP-GRs is distributed throughout the nucleoplasm of control cells, while accumulates in nucleoli upon siRNA-mediated knock-down of PRMT1. Altogether these results indicate that PRMT1-dependent methylation of PFV Gag C-terminal region requires the invariant R540 residue and is necessary to prevent Gag accumulation in nucleoli.

## Discussion

It is well established that incoming FV Gag tethers the PIC to host cell chromatin contributing to integration site selection [19–21]. We also showed that PFV Gag harbors a NES, which integrity is required for the completion of the late stages of viral replication [28]. These observations suggest that Gag transits through the nucleus at a step following its translation although the mechanisms underlying its ability to cross the nuclear membrane are still unclear [20]. To get further insights in the role of Gag nuclear trafficking for FV replication, we investigated the localization of the C-terminal GR boxes and found that GRI and GRIII act as NoLSs able to target a heterologous protein (GFP) to the nucleolus. This observation underscores once more the functional link between these two motifs [22]. We also provide evidence that PFV Gag accumulates to nucleoli in a context of infection using two complementary approaches. First, we demonstrated that Gag binds to and, at least partially, colocalizes with a Gag-TRAP-GFP decoy constitutively localized in the nucleolus of PFV-infected cells. Second, we visualized Gag in the nucleolus of PFV-infected cells exposed to conditions that slow down protein trafficking (hypoxia and/or LMB treatment), indicating that nucleolar accumulation occurs during the viral cycle and is not a mere artifact of Gag-TRAP-GFP expression. Our findings complement previous reports that Gag and/or the isolated NC proteins from several retroviruses display nuclear/nucleolar distribution, when expressed either as individual proteins or during viral infection (reviewed in [30]). The importance of this nuclear/nucleolar stage for retroviral replication is still unknown. Notably, the observation that many proteins of RNA viruses involved in genome packaging and viral particle assembly localize to nucleoli [44, 45] suggests that these nuclear bodies could be sites where viral ribonucleoprotein complexes form to facilitate viral RNA export and packaging [46].

Another major finding of our work is that neither full-length Gag nor an N-terminal truncation mutant encompassing the three GR boxes accumulates in nucleoli unless the entire GRII box or the CBS is deleted, leading us to assume that this region might hold determinant(s) antagonizing nucleolar targeting. We mapped this determinant to the invariant R540 residue, which mutation is sufficient to induce nucleolar targeting of the isolated GRII box or Gag CTD. Of note, localization of full-length Gag harboring the R540A mutation in nucleoli required concomitant inactivation of N-terminal motifs such as the CTRS, the NES or the dimerization domain, conditions that favor nuclear accumulation of the viral protein. Consistent with this observation, Müllers et al. [20] reported that PFV Gag displays nucleolar staining when fused to a heterologous NLS and upon simultaneous deletion of the GRII box. Similarly, Lochmann et al. [46] described nucleolar localization of RSV Gag after having enhanced its concentration in the nucleus by inhibition of CRM1-dependent nuclear export or mutation of its NES.

Having previously shown that the CBM mediates tethering of PFV Gag to chromatin [19], we assessed the implication of R540 in this process. Our data show that substitution of R540 to A prevents Gag from binding to mitotic chromosomes and interacting with the H2A histone. This finding is in agreement with the recent results of Lesbats et al. [21] demonstrating that R540 acts as an anchor motif interacting with the acidic patch on the surface of the H2A/H2B heterodimer. The role of R540 in modulating chromatin binding is further supported by the observation that insertion of the WT chromatin-binding sequence (CBS, aa 534–546) of PFV Gag, but not the corresponding R540A mutant, restores the interaction between a mutant version of MLV p12 and mitotic chromosomes [47]. Notably, we found that Gag CTD bearing the R540 K mutation associates to chromatin in cells undergoing unperturbed mitosis, indicating that a positive charge at position 540 is necessary and sufficient to ensure tethering of PFV Gag on chromatin, but not to antagonize nucleolar accumulation.

Finally we set to investigate how R540 regulates the subnuclear localization of PFV Gag. Post-translational modification of R by methylation has been shown to modulate the function and/or localization of several viral proteins. Studies on HIV NC revealed that mutation of R residues within the NoLSs, which are targeted by PRMT6-mediated methylation, impairs both nucleolar localization [46] and reverse transcription initiation [48]. Methylation is also proposed to control both the nucleolar distribution and the transactivation activity of the HIV Tat protein [49, 50]. In the case of HIV Rev mutation of methylated R residues or expression of catalytically inactive PRMT6 diminishes both binding to and nuclear export of RRE-containing transcripts [51]. Least but not last, methylation of R residues influences histone binding of KSHV (Kaposi Sarcoma-associated Herpesvirus) LANA (Latency-associated Nuclear Antigen) protein [52]. Based on these reports, we asked whether PFV Gag is methylated and whether this post-translational modification might influence its subnuclear distribution. In our work we show for the first time that PFV Gag interacts with and is methylated by both PRMT1 and PRMT5. We also established that Gag R540A mutant retains the ability to interact with PRMT5 and, surprisingly, displays enhanced SDMA modification compared to WT Gag. Why mutation of the R540 residue would facilitate deposition of SDMA marks by PRMT5 and at which sites this modification occurs are currently unanswered questions. Importantly, substitution of R540 with A abolished both Gag association with, and modification by, PRMT1. In addition, PFV Gag C-terminus fused to GFP (GFP-GRs) is enriched in nucleoli when PRMT1 expression is reduced by RNA interference, mimicking the phenotype of the R540 mutation. These data are consistent with a model according to which PRMT1-mediated modification of PFV Gag antagonize its nucleolar accumulation. Although we do not provide direct evidence of PRMT1-mediated methylation of R540, it is tempting to speculate that reversible modification of this amino acid might contribute to finely tune the distinct functions of Gag at different stages of the replication cycle. Nevertheless, finding that Gag mutants where R540 is mutated to A or F, which mimics constitutive methylation [53, 54], have a similar phenotype argues that methylation at this site is neither required for localization in nucleoli nor for tethering to chromatin. Another possibility is that PRMT1 controls Gag subnuclear localization by mediating ADMA modification of other residues within its R-rich C-terminal region, which await identification. PFV Gag was already known to be phosphorylated on multiple sites [5, 9]. Recent studies established that phosphorylation of T225 occurs exclusively in virions and propose that this modification is required for the interaction between PFV Gag and Polo-like kinases, ultimately leading to efficient integration [55].

When we assessed the impact of nucleolar retention of Gag on PFV replication we found that virions produced from Gag-TRAP-GFP-expressing cells display only a moderate decrease of infectivity, which is not statistically significant as compared to the infectivity of virions released from cells expressing Gag_1-200_-GFP, leaving the question of the role of PRMT1-dependent methylation and/or nucleolar accumulation of Gag during PFV replication open for further investigations. Given that a virus harboring the R540Q mutation within the CBM has an altered integration profile [21], it would also be interesting to address the role of Gag methylation for integration site selection.

## Conclusion

In closing, our work underscores that Gag localizes in the nucleolus during PFV replication. This step is likely regulated by PRMT1-mediated methylation of Gag that depends on the invariant R540 residue. Further studies will be required to define the functional significance of the nucleolar step for FV replication as well as the consequences of PFV Gag methylation in regard to the regulation of its complex nuclear transport and integration site selection.

## Methods

### Cells and culture conditions

HeLa, 293T and U373MG cells were cultured in DMEM supplemented with 10% Fetal Calf serum (FBS). BHK-U3GFP indicator FAG (Fluorescence Activated GFP) cells were cultured in DMEM supplemented with 5% FBS and 500 µg/mL G418 (Gibco). Leptomycin B (LMB) (Sigma) was added to culture medium to a final concentration of 10 nM for 4 h. Hypoxic conditions (2% O_2_, 5%CO_2_ and 93% N_2_) were induced by a continuous flow of nitrogen using a Forma Series II Water Jacket CO2 incubator (model: 3131; Thermo Scientific).

### Plasmid constructions

Fusion of individual GR boxes (GRI: aa 485–511, GRII: aa 534-557, GRIII: aa 586–618) or RevNoLS (aa 35–51) to GFP or RFP was obtained by inserting annealed complementary oligonucleotides of appropriate sequence into pEGFP-C1 or pDsRed-C1. GFP-GRs (GRs: aa 477–625) and GFP-Gag were constructed by insertion of PCR products obtained using the pcziGag as template, into pEGFP-C1 (Clontech) between HindIII and BamHI sites. HA-GRs expression plasmids were generated by replacing GFP by the HA sequence. pHFVGag∆_131-162_ [39] served as template to generate GFP-Gag∆dim (deletion of PFV Gag aa 130–160). Mutants were generated using QuickChange site-directed mutagenesis Kit according to the manufacturer’s specifications (Stratagene). Fragments spanning Gag_1-200_, RevNoLS or Gag_1-200_ fused to RevNoLS, were inserted into pEGFP-N1, and the resulting plasmids were used as template to amplify the coding sequences to be inserted in pMSCVneo at the EcoRI and HpaI sites. Plasmids encoding GFP-tagged human PRMTs proteins were kindly provided by Mark Bedford [56].

### Establishment of cell lines stably expressing Gag-TRAP-GFP

U373MG stable cell lines were established using the Murine Stem Cell Virus (MSCV)-based retroviral vector system. Recombinant retroviral vectors were generated by transfection of 293T cells with the pMSCV-neo vector encoding Gag_1-200_-RevNoLS-GFP (Gag-TRAP-GFP), Gag_1-200-_GFP, RevNoLS-GFP or GFP, and the packaging plasmids expressing MLV Gag-Pol and the Vesicular Stomatitis Virus envelope G glycoprotein (VSV-G) using the calcium phosphate precipitation method. Cell-free supernatants were collected 48 h post-transfection and used to transduce U373MG cells. GFP expression was analyzed 48 h post-transduction by flow cytometry. After cell sorting, GFP-positive cells were propagated in culture medium supplemented with G418 (500 µg/mL).

### Immunocytochemistry

Indirect immunofluorescence imaging on fixed-cells or mitotic chromosome spreads was described elsewhere [19]. In brief, samples were incubated with the appropriate primary antibodies (4 °C, overnight) and fluorescent-labeled secondary antibody (30 min, room temperature). Nuclei were stained with 4,6-diamidino-2-phenylindole (DAPI). Images were acquired with a laser-scanning confocal microscope (LSM510 Meta; Carl Zeiss) equipped with an Axiovert 200 M inverted microscope, using a Plan Apo 63_/1.4-N oil immersion objective.

### Co-immunoprecipitation assay and Western blotting

Cell pellets were lysed in 0.4 M NaCl, 1 mM MgCl_2_, 10% sucrose, 0.5 mM DTT, 10 mM PIPES pH 6.8, 0.5% NP-40 supplemented with Protease Inhibitor Cocktail (Roche) (30 min on ice), and subsequently centrifuged (12,000*g*, 5 min at 4 °C). Immunoprecipitation and Western-blot were performed as previously described [28].

### Immuno-electron microscopy

Transfected 293T cells were prepared as described [57]. After extensive washing, the grids were incubated with an anti-GFP monoclonal antibody (90 min, RT), followed by an anti-rabbit antibody conjugated to 15 nm-gold particles (British Biocell International, Cardiff, UK) (90 min, RT). Ultrathin sections were stained with 5% uranyl acetate 5% lead citrate, placed on EM grids coated with collodion membrane and observed with a Jeol 1010 transmission electron microscope (Tokyo, Japan).

### RNA interference

HeLa Cells were transiently transfected with ON-TARGETplus Human SMARTpool siRNA targeting PRMT-1 (Dharmacon #3276) or the scrambled control (10 nM) using the Lipofectamine RNAiMax reagent according to the manufacturer’s instructions (Life Technologies). After 48 h, cells were transfected with the GFP-GRs expressor and, following further 24 h incubation, were fixed and analyzed by confocal microscopy.

### Statistic testing

Graphical representation and statistical analyses were performed using the GraphPad Prism software (GraphPad Software, San Diego, CA, USA). Differences were tested for statistical significance using ne-way ANOVA statistical test with a Bonferroni Multiple comparison post-test.

## Additional files

**Additional file 1: Figure S1.** Nucleolar targeting is a conserved feature of EFV GRI and GRIII boxes and is antagonized by R472 within GRII. **A)** Amino acid sequences of the GR boxes of PFV and EFV and the NoLS of HIV-1 Rev protein (aa 35–51). **B)** Electron microscopy images of HeLa cells expressing GFP, GFP-GRI or GFP-RevNoLS and stained with an anti-GFP antibody (ab6556, Abcam, 1:200) and a secondary antibody coupled to 15 nm gold particles (goat anti-rabbit 15 nm Gold, BBI International, 1: 60). **C)** PFV GRI fused to DsRed and PFV GRIII, EFV GRI (aa 395–427) or GRIII (aa 492–524) fused to GFP were expressed in HeLa cells. Their localization was analyzed 24 h later as described in Fig. 1b. Nuclei are stained with DAPI. **D)** The C-terminal region (GRs) of EFV Gag fused to GFP and bearing the R472A mutation or not, was expressed in HeLa cells, and its localization was studied as described in Fig. 1b. Nuclei are stained with DAPI. Scale bar represents 10 µm.

**Additional file 2: Figure S2.** Gag-TRAP-GFP interacts with PFV Gag. Lysates of 293T cells ectopically expressing PFV Gag and Gag-TRAP-GFP construct (Gag_1-200_-RevNoLS-GFP) or the corresponding controls (GFP, Gag_1-200_-GFP or RevNoLS-GFP) were immunoprecipitated on protein A beads coated with an anti-GFP antibody (cat.11 814 460 001, Roche, 1:100). Input and bound proteins were analyzed as in Fig. 4a.

**Additional file 3: Figure S3.** Both WT and R540A mutant Gag bind to PRMT5. **A)** Lysates from 293T cells expressing PFV Gag and each human PRMT variant in fusion with GFP were immunoprecipitated with protein A beads coated with anti-PFV antibodies. Input and bound proteins were analyzed as in Fig. 4a. **B)** Cells expressing WT or R450A mutant PFV Gag and GFP-PRMT5 were lysed and incubated with beads coated with anti-GFP (cat.11 814 460 001, Roche, 1:100) or anti-SDMA (SYM10, 07-412, Millipore, 1:100) antibodies. Input and immunoprecipitated proteins were treated as in Fig. 4a.
