# Supplementary figures and images for: The invariant arginine within the chromatin-binding motif regulates both nucleolar localization and chromatin binding of Foamy virus Gag
Source: Retrovirology. 2018 Jul 11;15:48. doi: 10.1186/s12977-018-0428-z (PMC6042332; doi:10.1186/s12977-018-0428-z)

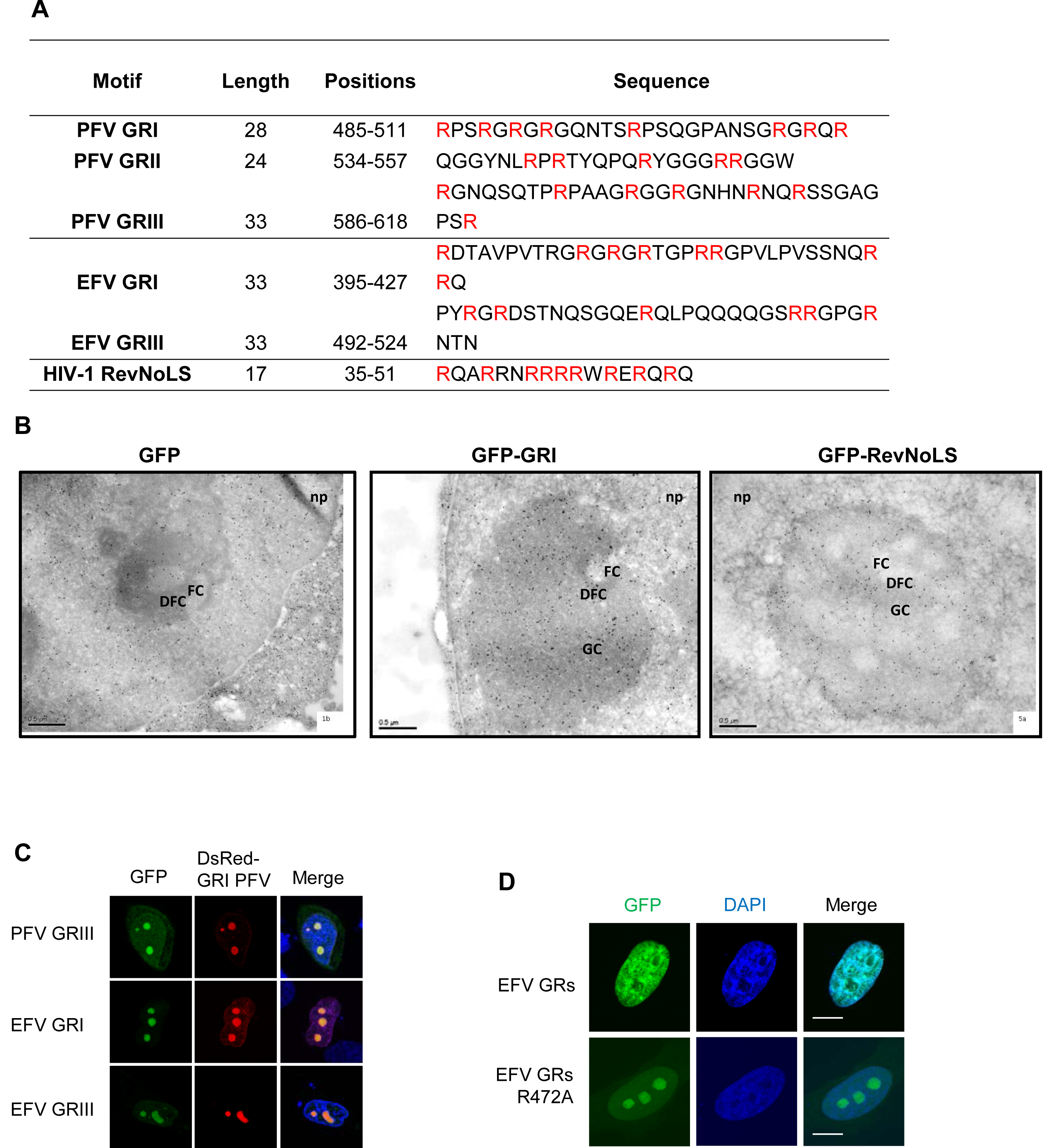

Supplement: Supplementary file 1 — Additional file 1: Figure S1. Nucleolar targeting is a conserved feature of EFV GRI and GRIII boxes and is antagonized by R472 within GRII. A) Amino acid sequences of the GR boxes of PFV and EFV and the NoLS of HIV-1 Rev protein (aa 35–51). B) Electron microscopy images of HeLa cells expressing GFP, GFP-GRI or GFP-RevNoLS and stained with an anti-GFP antibody (ab6556, Abcam, 1:200) and a secondary antibody coupled to 15 nm gold particles (goat anti-rabbit 15 nm Gold, BBI International, 1: 60). C) PFV GRI fused to DsRed and PFV GRIII, EFV GRI (aa 395–427) or GRIII (aa 492–524) fused to GFP were expressed in HeLa cells. Their localization was analyzed 24 h later as described in Fig. 1b. Nuclei are stained with DAPI. D) The C-terminal region (GRs) of EFV Gag fused to GFP and bearing the R472A mutation or not, was expressed in HeLa cells, and its localization was studied as described in Fig. 1b. Nuclei are stained with DAPI. Scale bar represents 10 µm. [file 12977_2018_428_MOESM1_ESM.tif]

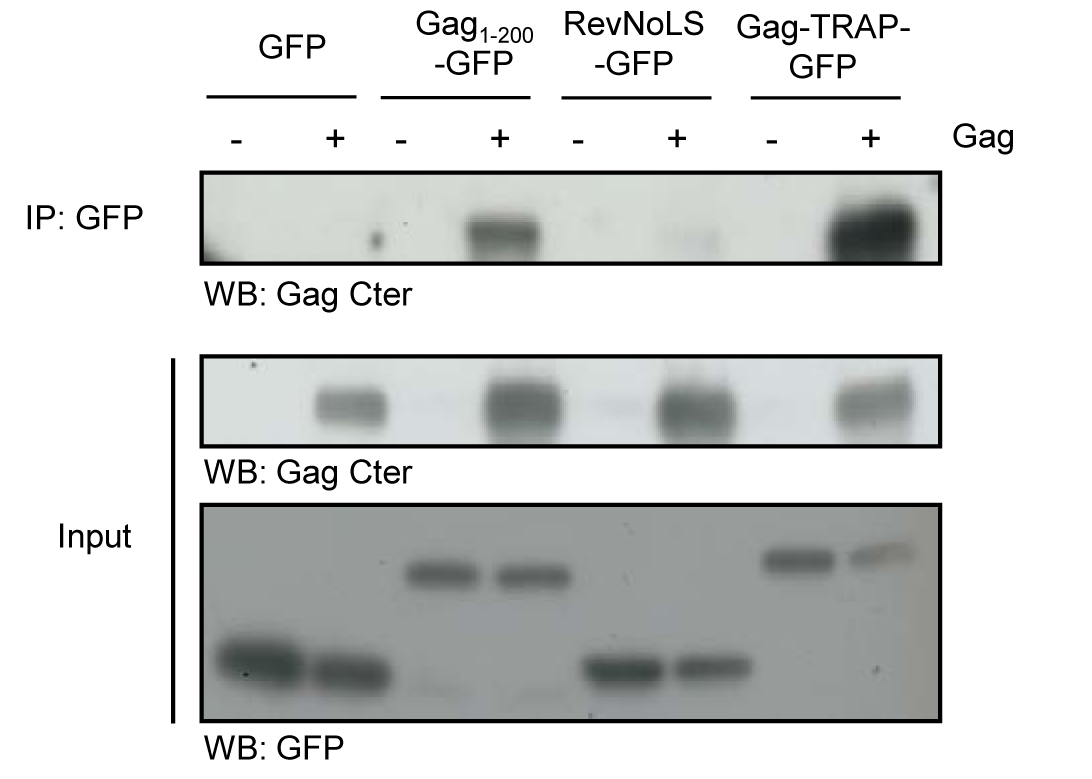

Supplement: Supplementary file 2 — Additional file 2: Figure S2. Gag-TRAP-GFP interacts with PFV Gag. Lysates of 293T cells ectopically expressing PFV Gag and Gag-TRAP-GFP construct (Gag1-200-RevNoLS-GFP) or the corresponding controls (GFP, Gag1-200-GFP or RevNoLS-GFP) were immunoprecipitated on protein A beads coated with an anti-GFP antibody (cat.11 814 460 001, Roche, 1:100). Input and bound proteins were analyzed as in Fig. 4a. [file 12977_2018_428_MOESM2_ESM.tif]

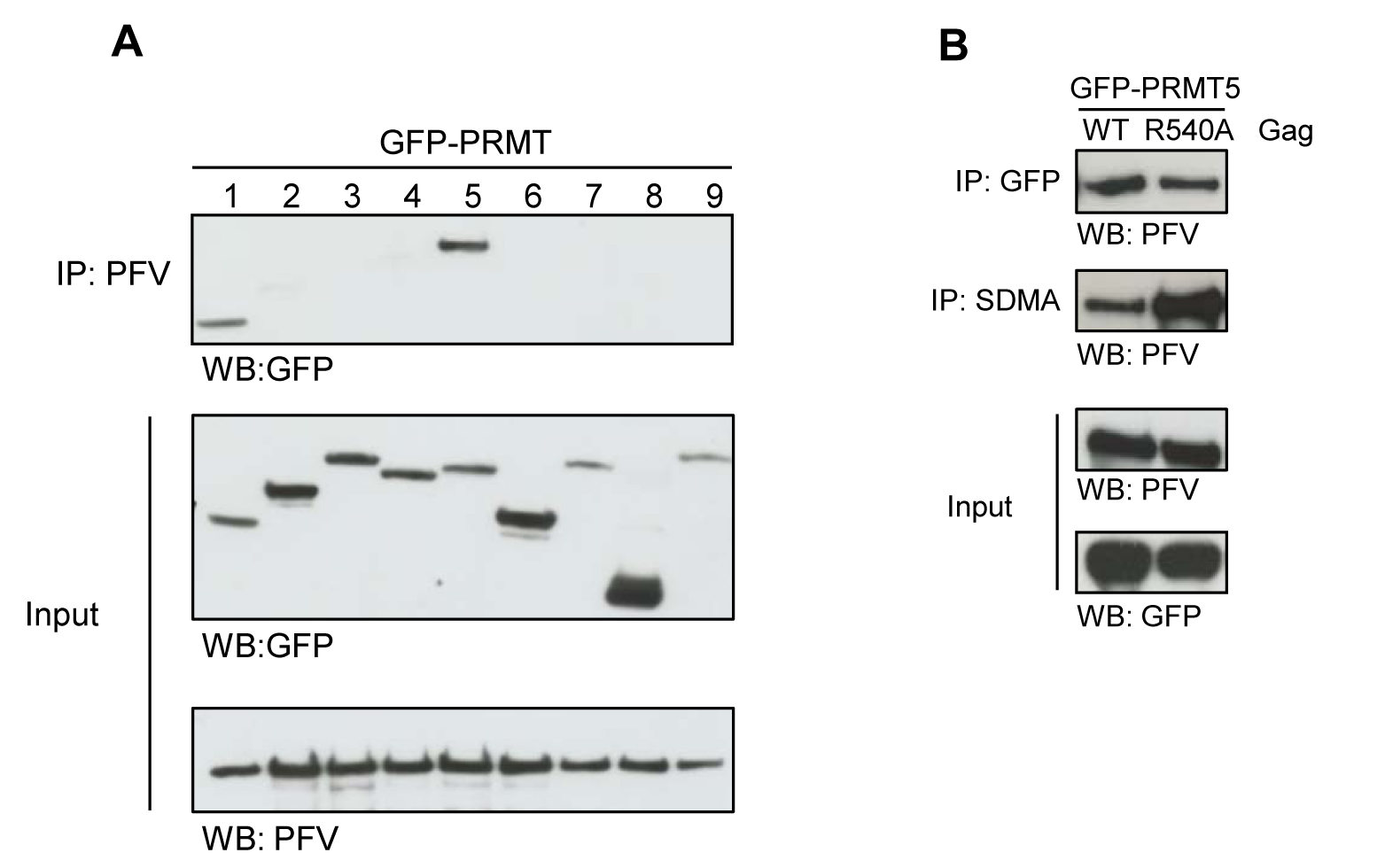

Supplement: Supplementary file 3 — Additional file 3: Figure S3. Both WT and R540A mutant Gag bind to PRMT5. A) Lysates from 293T cells expressing PFV Gag and each human PRMT variant in fusion with GFP were immunoprecipitated with protein A beads coated with anti-PFV antibodies. Input and bound proteins were analyzed as in Fig. 4a. B) Cells expressing WT or R450A mutant PFV Gag and GFP-PRMT5 were lysed and incubated with beads coated with anti-GFP (cat.11 814 460 001, Roche, 1:100) or anti-SDMA (SYM10, 07-412, Millipore, 1:100) antibodies. Input and immunoprecipitated proteins were treated as in Fig. 4a. [file 12977_2018_428_MOESM3_ESM.tif]
